# When less is more: deprescription in a long-stay hospital. A case report

**DOI:** 10.1192/j.eurpsy.2022.1882

**Published:** 2022-09-01

**Authors:** R. De Hita Santillana, A. Cerame, M.L. Costa

**Affiliations:** 1 Hospital Universitario José Germain, Ucpp, Leganés, Spain; 2 Hospital Universitario José Germain, Psychiatry Department, Leganés, Spain; 3 Hospital Universitario José Germain, Hospital De Día, Leganes, Spain; 4 Hospital Universitario Severo Ochoa, Psychiatry, Leganes, Spain

**Keywords:** Side effects, deprescription, polymedication

## Abstract

**Introduction:**

We present the case of a 54-year-old man diagnosed with mixed histrionic / obssesive personality disorder admitted in a long-stay hospital. Symptoms began at 40 years of age, predominantly anxiety and agoraphobia alongside somatic complaints. During his admission, the symptoms markedly fluctuated. The patient alternated periods in which he presented confusion with others of irritability, disinhibition, and stereotyped movements. Moreover the patient spent long periods of time in bed with little or no communication with other patients or staff.

The different pharmacological approaches which were carried out and their consequences are analyzed.

**Objectives:**

It is a common practice to increase the number of prescribed drugs or their doses when symptoms worsen. The result is polymedication and higher doses above maximum levels in the technical sheet. Reducing medication is rarely considered as a strategy.

**Methods:**

A case report is presented alongside a review of the relevant literature regarding different long-term pharmacological treatments and their side effects.

**Results:**

The suspension of the antipsychotic treatment which had been administered for years represented a significant improvement. Withdrawal of Olanzapine resulted in a significant improvement.

**Conclusions:**

It is important to review the prescription of each medication in time, as well as to consider their possible side effects.

**Disclosure:**

No significant relationships.

